# Enzymatic Preparation and Processing Properties of DPP-IV Inhibitory Peptides Derived from Wheat Gluten: Effects of Pretreatment Methods and Protease Types

**DOI:** 10.3390/foods13020216

**Published:** 2024-01-10

**Authors:** Rui Zhao, Shuwen Lu, Shaozhen Li, Huifang Shen, Yao Wang, Yang Gao, Xinting Shen, Fei Wang, Jiawu Wu, Wenhui Liu, Kaixin Chen, Xinmiao Yao, Jian Li

**Affiliations:** 1 Key Laboratory of Green and Low-Carbon Processing Technology for Plant-Based Food of China National Light Industry Council, Beijing Technology and Business University, No. 33 Fucheng Road, Beijing 100048, China; lilyamongthorns@163.com; 2 Food Processing Research Institute, Heilongjiang Academy of Agricultural Sciences, Harbin 150086, China; shuwenl@hass.cn (S.L.); 13904648796@163.com (H.S.); wang1221yao1221@163.com (Y.W.); gao20031026@163.com (Y.G.); 15663585599@163.com (X.S.); wangf2022822@163.com (F.W.); wujiawu1115@163.com (J.W.); 4228041@163.com (K.C.); 3 Beijing Huiyuan Food & Beverage Co., Ltd., Beijing 101305, China; li.shaozhen@huiyuan.com.cn (S.L.); liu.wenhui@huiyuan.com.cn (W.L.); 4 Heilongjiang Province Key Laboratory of Food Processing, Harbin 150086, China; 5 Heilongjiang Province Engineering Research Center of Whole Grain Nutritious Food, Harbin 150086, China

**Keywords:** DPP-IV inhibitory peptide, pretreatment, enzymatic hydrolysis performance, processing properties

## Abstract

The choice of appropriate proteases and pretreatment methods significantly influences the preparation of bioactive peptides. This study aimed to investigate the effects of different pretreatment methods on the hydrolytic performance of diverse proteases during the production of dipeptidyl peptidase-IV (DPP-IV) inhibitory peptides derived from wheat and their foaming and emulsion properties. Dry heating, aqueous heating, and ultrasound treatment were employed as pretreatments for the protein prior to the enzymatic hydrolysis of wheat gluten. FTIR analysis results indicated that all pretreatment methods altered the secondary structure of the protein; however, the effects of dry heating treatment on the secondary structure content were opposite to those of aqueous heating and ultrasound treatment. Nevertheless, all three methods enhanced the protein solubility and surface hydrophobicity. By using pretreated proteins as substrates, five different types of proteases were employed for DPP-IV inhibitory peptide production. The analysis of the DPP-IV inhibitory activity, degree of hydrolysis, and TCA-soluble peptide content revealed that the specific pretreatments had a promoting or inhibiting effect on DPP-IV inhibitory peptide production depending on the protease used. Furthermore, the pretreatment method and the selected type of protease collectively influenced the foaming and emulsifying properties of the prepared peptides.

## 1. Introduction

Type II diabetes mellitus is a metabolic disorder characterized by impaired insulin metabolism, which often leads to significant suffering for patients due to its acute complications [1]. Insulin, the sole plasma hormone responsible for reducing glycemic levels, plays a vital role in maintaining glucose homeostasis [2]. The secretion of insulin can be enhanced through the stimulation of glucose-dependent insulinotropic polypeptide and glucagon-like peptide-1 [3]; however, their degradation by dipeptidyl peptidase-IV (DPP-IV) occurs rapidly under physiological conditions. Consequently, blocking the degradation of insulin has emerged as a strategy for the management of type II diabetes [4]. By inhibiting DPP-IV activity, DPP-IV inhibitors can extend the half-life of glucose-dependent insulinotropic polypeptide and glucagon-like peptide-1, resulting in increased postprandial insulin secretion that facilitates better glycemic regulation [5]. Synthetic DPP-IV inhibitors like sitagliptin and vildagliptin are drugs that have demonstrated efficacy in treating type II diabetes.

DPP-IV inhibitory peptides are typically composed of no more than 20 amino acids [3]. Numerous studies have demonstrated that *in-vitro*-validated DPP-IV inhibitory peptides often possess hypoglycemic effects *in vivo* [6,7]. Furthermore, compared to synthetic drugs, naturally derived DPP-IV inhibitors from food sources are generally considered safe and can be utilized as functional ingredients in food production [8]. *In silico* approaches have been employed to assess the potential of various dietary proteins as precursors for the generation of DPP-IV inhibitory peptides. The findings revealed wheat gluten protein (WGP) to be one of the most promising sources for such peptides [9,10].

The enzymatic hydrolysis of dietary proteins offers several advantages, including high efficiency, a controllable process, strong specificity, mild conditions, and no pollution. It is the most commonly employed method of releasing DPP-IV inhibitory peptides [7,11]. However, the enzymatic hydrolysis of WGP is often hindered by the masking of enzyme cleavage sites and the protein’s poor solubility, which can be attributed to the high content of hydrophobic groups within the protein [12]. Consequently, both the efficiency of enzymatic hydrolysis and the protein conversion rate of WGP are relatively low [13].

It has been demonstrated that many physical treatments can induce protein denaturation, which facilitates protease–protein accessibility by unfolding protein structures. For example, the application of ultrasound treatment induces cavitation and ultrasonic mechanical effects, which result in enhanced enzymatic efficiency by exposing interior enzymolysis sites, reducing the hydrolysis time, and increasing the degree of hydrolysis (DH) [13,14]. Additionally, appropriate heating can also alter the spatial structure of proteins, exposing sites for enzymolysis. This further enhances the DH and has the potential to increase both the quantity and quality of the generated peptides [15].

It is worth noting that these pretreatment conditions should be carefully optimized to avoid excessive protein denaturation, which may result in refolding and the embedding of enzyme cleavage sites, thereby adversely affecting enzymatic hydrolysis. Furthermore, pretreatments can potentially modify the processing properties of proteins. However, limited research has been conducted on the processing properties of DPP-IV inhibitory peptides thus far; this aspect deserves attention when considering their utilization as functional food ingredients. Moreover, there is a paucity of studies investigating the effects of different pretreatment methods on the enzymatic hydrolysis performance of diverse proteases.

The purpose of this study was to investigate the effects of different pretreatment methods on the enzymatic hydrolysis properties of various types of proteases and the processing properties of hydrolysates. The main research content included several aspects as follows: (1) the impact of different pretreatment methods on protein secondary structure, solubility, and surface hydrophobicity; (2) the influence of different pretreatment methods on the hydrolytic performance of various proteases, including the hydrolysis degree, biological activity, and TCA-soluble peptide content; (3) the foaming properties and emulsion activity of enzymatic products.

## 2. Materials and Methods

### 2.1. Materials

WGP was purchased from Huaxin Co., Henan, China. The gluten contained 82.9% (*w*/*w*, dry basis) protein and 7.2% moisture. The commercial enzymes used in this study were ProteAXH (≥1600 U/g, Amano Enzyme Inc., Nagoya, Japan), Protamex^®^ 1.6 (1.6 AU-N/g, Novozymes A/S, Bagsvaerd, Denmark), Flavourzyme^®^ 500 MG (500 LAPU/g, Novozymes A/S, Bagsvaerd, Denmark), neutral protease (50,000 U/g, Solarbio LIFE SCIENCES, Beijing, China), and acid protease (50,000 U/g, SUNSON Industry Group Co., Ltd, Beijing, China). Recombinant human DPP-IV (>20 U/mg) was obtained from ProSpec-Tany TechnoGene Ltd., Ness-Ziona, Israel. All other chemicals used in the study were of analytical grade.

### 2.2. Pretreatment Methods

Three individual methods were used for the pretreatment of WGP before its hydrolysis. Their processing conditions were as follows.

Dry heating treatment (DHT): WGP was evenly tiled on a plate and baked at 85 °C for 30 min, with stirring once during this time.

Aqueous heating treatment (AHT): WGP was dispersed in distilled water at 1:10 (*w*/*v*) and held at 85 °C for 30 min in a water bath. The dispersion was continuously stirred with a two-blade agitator. At the end of DHT and AHT, the samples were immediately cooled to ambient temperature.

Ultrasound treatment (UST): WGP was dispersed in distilled water at 1:10 (*w*/*v*) and subjected to ultrasound treatment at 400 W for 20 min, with intermittent 4 s every 8 s.

Untreated WGP served as the control. The samples subjected to AHT and UST were freeze-dried and ground into powders. All the samples were kept sealed at −20 °C.

### 2.3. Fourier Transform Infrared Spectroscopy Determination

The Fourier transform infrared spectroscopy (FTIR) spectra of WGP were measured according to the method described by Liu et al. [16]. The Omnic 6.0 and PeakFit 4.12 software were applied to analyze the peaks of the amide I region (1600 to 1700 cm^−1^) by Fourier deconvolution and second derivative analysis. The absorption peaks of corresponding secondary structures were assigned according to previous studies [17,18].

### 2.4. Soluble Protein Content Determination

The soluble protein content was determined according to the method used by Zhang et al. [19], with minor modifications. First, 10 mg/mL of WGP was prepared in 0.01 M phosphate buffer (pH 7.0) and mixed thoroughly under magnetic stirring at room temperature for 30 min. After centrifugation at 10,000× *g* for 20 min, the protein content in the supernatant (mg/mL) was determined by the BCA method, with the standard curve prepared using bovine serum albumin.

### 2.5. Surface Hydrophobicity

The surface hydrophobicity (*H*_0_) of WGP was measured by the hydrophobic chromophore bromophenol blue solution (BPB) method [20], with slight modifications. This method can be applied in the determination of the *H*_0_ of non-soluble proteins [21].

The WGP suspension (1 mL, 5 mg/mL in 20 mM phosphate buffer, pH 6.5) was thoroughly mixed with 200 µL of BPB (1 mg/mL in distilled water). Here, 1 mL of phosphate buffer instead of the WGP suspension was used as the blank control. The samples and the blank control were continuously stirred at room temperature for 10 min and then centrifuged at 2000 r/min for 15 min. Then, 300 µL of the supernatant was diluted 10 times with the buffer, and the absorption value was read at 595 nm.

(1) $$BPB\;bound\;(µg)=200\times \frac{A_{blank}-A_{sample}}{A_{blank}}$$

where A: the absorbance at 595 nm

### 2.6. Preparation of WGP Hydrolysates

An 8% (*w*/*v*) aqueous dispersion of the WGP preparation was incubated in a water bath at 55 °C for 30 min. The pH of the solution was adjusted to 7.0 with NaOH for ProteAXH, Protamex, Flavourzyme, and the neutral protease and 3.6 with citric acid for the acid protease, respectively. Hydrolysis was carried out with an enzyme-to-substrate ratio of 1:100 (*w*/*w*) at 55 °C for 4 h under agitation. After hydrolysis, proteases were inactivated by heating in a water bath at 95 °C for 10 min. After cooling to room temperature, the hydrolysates were centrifuged at 10,000× *g* for 10 min and the supernatants were frozen, freeze-dried, and stored at −20 °C for further analysis.

### 2.7. Degree of Hydrolysis Determination

The degree of hydrolysis (DH) was expressed as the percentage increase in the amount of α-amino groups, which were generated when peptide bonds were broken by the protease. The content of free amino nitrogen was measured using the o-phthaldialdehyde (OPA) method [22].

Briefly, a 32 μL aliquot of the diluted sample was mixed with 240 μL of freshly prepared OPA reagent and absorbance values at 340 nm were read after incubation at room temperature for 2 min. The standard curve was prepared using serine solution (0 to 0.97 mM). The DH values were calculated as in the following equation:

(2) $$DH\;(\%)=\frac{S-C}{T}\times 100$$

where S represents the reactive α-amino groups in the hydrolysate of WGP, C represents the reactive α-amino groups in unhydrolyzed WGP, and T represents the total number of amino groups found in native WGP, which was determined by subjecting it to acid hydrolysis using 6 M HCl at 110 °C for 24 h.

### 2.8. TCA-Soluble Peptide Content

The content of TCA-soluble peptides was determined using the method described by Zhu et al. [23]. The hydrolysate was 10-fold diluted with 5% TCA solution (*w*/*w*), vortexed for 30 s, and maintained at ambient temperature for another 30 min. After centrifugation (10,000× *g* for 10 min, 4 °C), the peptide content in the supernatant was determined by a BCA test kit.

(3) $$TCA\;soluble\;peptide\;content\;(\%)=\frac{\mathrm{peptide}\;\mathrm{content}\;\mathrm{in}\;\mathrm{supernant}}{\mathrm{total}\;\mathrm{protein}\;\mathrm{content}}\times 100$$

### 2.9. DPP-IV Inhibition Assay

The DPP-IV inhibition assay was carried out according to the method outlined by Nongonierma and FitzGerald [5], with slight modifications. In short, 25 μL of the sample was mixed with 50 μL of the reaction substrate GP-pNA (final concentration of 0.200 mM). The reaction was initiated by adding 50 μL of DPP-IV (final concentration of 2.5 mU/mL). All the reagents were prepared using Tris–HCl buffer (100 mM, pH 8.0). An equivalent volume of Tris–HCl buffer instead of the sample was employed as the negative control. After incubation at 37 °C for 30 min in a microplate reader (BioTek Synergy HT, Bio-Tek Instruments Inc., Winooski, VT, USA), the absorbance was monitored at 405 nm.

### 2.10. Foaming Properties

The foaming capacity (FC) and foam stability (FS) were determined following the method described by Zielińska et al. [24], with minor modifications. Foams were prepared by blending 10 mL of WGP hydrolysate (1 mg/mL) for 120 s at a high speed of 15,600 r/min using an IKA^®^ ULTRA-TURRAX^®^ T 18 basic (IKA Works GmbH & Co., Staufen, Germany). The volume was recorded at time 0 and 30 min after homogenization. FC and FS were calculated as in the following formulas:

(4) $$FC\;(\%)=\frac{V_{0}-V}{V_{0}}\times 100$$

(5) $$FS\;(\%)=\frac{V_{30}}{V_{0}}\times 100$$

where V—the volume of the hydrolysate solution (10 mL), V_0_—the volume after whipping at time 0 min, V_30_—the remaining foam volume after standing for 30 min.

### 2.11. Emulsion Activity

The emulsion activity (EA) of the hydrolysates was determined according to the method used by Zielińska et al. [24]. The sample (1% in distilled water) was mixed with an equivalent volume of vegetable oil and homogenized at a speed of 20,000 r/min for 60 s. After centrifugation at 3000× *g* for 5 min, the volume of each layer was read. EA was calculated as in the following formula:

(6) $$EA\;(\%)=\frac{V_{e}}{V}\times 100$$

where V represents the total volume of the tube content and V_e_ is the volume of the emulsified layer.

### 2.12. Statistical Analysis

The experiments were independently replicated at least three times, and the data were presented as the mean ± standard deviation. One-way ANOVA was used to analyze the structure and physicochemical properties of WGP, while two-way ANOVA was employed to analyze the hydrolytic performance and processing properties of the resulting hydrolysates. Walter–Duncan’s test (*p* ≤ 0.05) was employed to determine significant differences between means. The statistical analysis was performed using the SPSS 21.0 software.

## 3. Results and Discussion

### 3.1. Structure and Physicochemical Properties

#### 3.1.1. Secondary Structure

**Figure 1:**
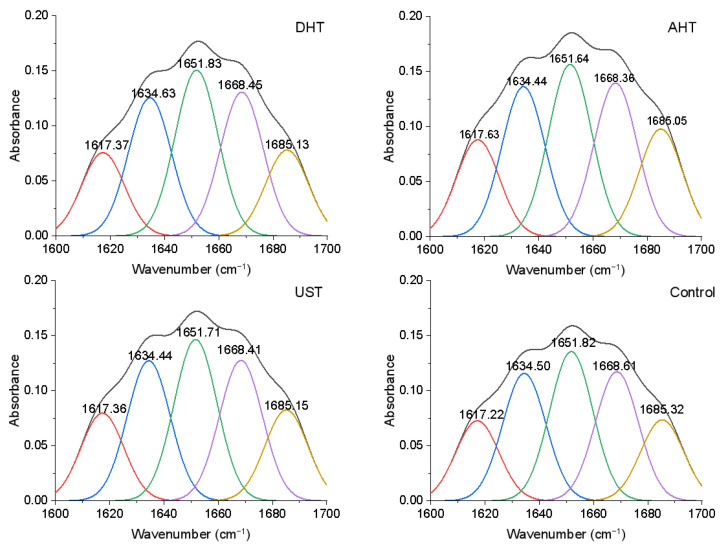
Secondary structure analysis of natural and pretreated wheat gluten proteins using FTIR spectroscopy. DHT: dry heating treatment; AHT: aqueous heating treatment; UST: ultrasound treatment.

FTIR spectroscopy was utilized to examine the variations in the secondary structures of WGPs resulting from diverse pretreatment methods. The amide I bands (1600 to 1700 cm^−1^) correspond to the stretching vibration of the C=O bond in the amide group [17], providing crucial insights into the secondary structures of proteins. By performing curve fitting on these spectra, a comprehensive understanding of the secondary structure of the WGPs was obtained, as depicted in Figure 1.

The bands observed at approximately 1617 (red), 1634 (blue), and 1685 cm^−1^ (yellow) are attributed to the β-sheet conformation. The prominent peak detected at 1651 cm^−1^ (green) is indicative of the α-helical conformation, although the possibility of an unordered conformation cannot be completely ruled out. The band located at 1668 cm^−1^ (purple) is assigned to the β-turn structure [18]. Detailed information regarding the assignment of deconvoluted bands and the proportions of secondary structures in WGPs subjected to different pretreatment methods can be found in Table 1.

The β-sheet was identified as the predominant secondary structure in WGPs, which was consistent with previous reports [18,25]. All pretreatments induced changes in the secondary structure content of WGP. Compared to the untreated control, DHT significantly increased the content of α-helix and β-turn (from 26.33% to 26.88% and from 22.78% to 23.30%, respectively, *p* < 0.05) and decreased the β-sheet content (from 50.89% to 49.82%, *p* < 0.05). Consequently, DHT led to a significant enhancement in the α-helix/β-sheet ratio (*p* < 0.05). In contrast, AHT and UST induced a transition from α-helix and β-turn to β-sheet, showing significant differences in the α-helix and β-sheet content, as well as their ratio, compared with the control group (*p* < 0.05). Therefore, AHT and UST caused structural changes in WGP that were completely opposite to those caused by DHT, with AHT having a greater impact on the structure of WGP than UST.

The observed structural changes of WGPs with different pretreatments were consistent with previous studies. Raman spectra were employed to analyze the modifications in the secondary structure of wheat protein following DHT. Quantitative analysis revealed that after DHT at 121 °C for 80 min, there was an increase in both the α-helix content and the α-helix to β-sheet ratio [26]. Furthermore, the high-intensity UST of WGP led to a reduction in α-helix content and the α-helix to β-sheet ratio [18]. The slight variations in protein secondary structure content observed in this study can be attributed to the gentle pretreatment conditions employed. Nevertheless, these findings are reasonable and may reflect disparities in the protein secondary structure changes caused by the different pretreatment methods.

#### 3.1.2. Soluble Protein Content and Surface Hydrophobicity

**Figure 2:**
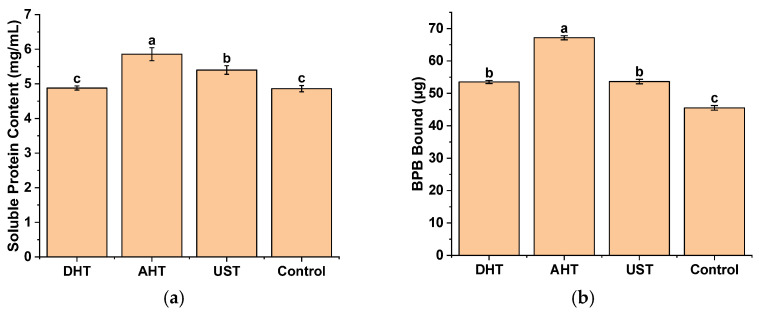
Physical properties of wheat gluten proteins with different pretreatments. (**a**) Soluble protein content; (**b**) surface hydrophobicity. DHT: dry heating treatment; AHT: aqueous heating treatment; UST: ultrasound treatment. The letters represent significant differences (*p* < 0.05).

Figure 2 illustrates the soluble protein content and surface hydrophobicity (*H*_0_) of WGPs with or without pretreatment. Although different effects on the secondary structure were observed, all treatment methods resulted in the improved solubility and *H*_0_ of WGPs.

The solubility of WGPs was significantly increased by 20.58% and 11.11% following AHT and UST, respectively (*p* < 0.05). However, the solubility of WGP treated with DHT showed no significant difference compared to the control (*p* > 0.05). All three pretreatment methods led to a substantial enhancement in the *H*_0_ of WGPs, with DHT, AHT, and UST increasing it by 17.58%, 47.55%, and 17.82%, respectively (*p* < 0.05).

Contrary to the expectation that a high *H*_0_ would lead to decreased solubility due to protein aggregation and precipitation, this study’s findings suggest that there is no necessary negative correlation between these two properties. All three pretreatment methods employed in this study were found to enhance the solubility and *H*_0_ of WGPs. Similar observations have been reported in which multi-frequency UST resulted in a simultaneous increase in the solubility of the WGP and its *H*_0_ [19].

Changes in protein structure can result in modifications to the distribution of hydrophobic groups on the protein surface, thereby impacting its solubility and propensity for aggregation or precipitation [27,28]. However, apart from *H*_0_, solubility is also influenced by the hydrophilic and hydrophobic properties of the proteins in the solution, which are affected by factors such as the protein structure and solvent composition [29,30]. In certain cases, there exists a positive correlation between protein solubility and *H*_0_ [28].

These findings indicate that the pretreatments utilized in this study induced the unfolding of the WGP structure and alteration of exposed amino acid sites, potentially influencing the protein’s enzymatic hydrolysis performance. However, the specific peptide segments affected by each treatment may vary; therefore, the impact of different treatment methods on the proteolytic hydrolysis of WGPs would differ.

### 3.2. Proteolysis Properties

#### 3.2.1. Degree of Hydrolysis

**Figure 3:**
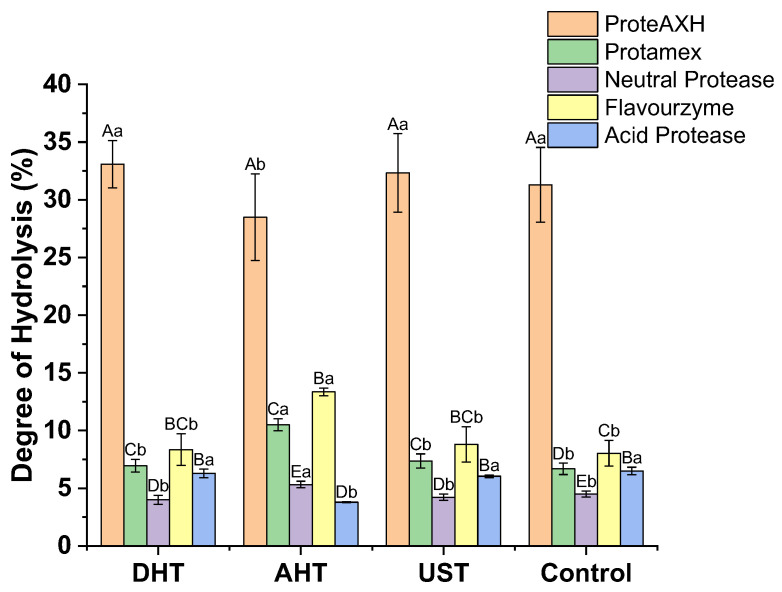
The degree of hydrolysis in the hydrolysates derived from wheat gluten proteins subjected to different pretreatments. DHT: dry heating treatment; AHT: aqueous heating treatment; UST: ultrasound treatment. Uppercase letters indicate significant differences within different pretreatments, while lowercase letters indicate significant differences within varied proteases (*p* < 0.05).

The WGPs, with or without pretreatment, were used as substrates and subsequently hydrolyzed separately by five distinct proteases. The degree of hydrolysis (DH) of the resulting hydrolysates is illustrated in Figure 3.

A two-way ANOVA was conducted to examine the influence of the protease type and pretreatment method on DH. The results revealed a significant effect of the protease type on DH (*p* < 0.01). Specifically, ProteAXH hydrolysis resulted in significantly higher DH values (28.48~33.09%) compared to other proteases (*p* < 0.01). The neutral protease exhibited the lowest DH, with a mean difference of −24.48% compared to ProteAXH. While the acid protease did not show a significant difference in its impact on DH compared to Flavourzyme, there were significant differences observed among different types of proteases (*p* < 0.01). The pretreatment methods did not demonstrate any significant differences in DH (*p* > 0.05), indicating that pretreatment alone does not affect DH. There was a significant interaction between the proteases and pretreatment methods (*p* < 0.01), suggesting that their combination has an important impact on DH outcomes. After AHT, the neutral protease, Protamex, and Flavourzyme led to a significant increase in DH values (*p* < 0.05), while the acidic protease resulted in a significant decrease in DH values (*p* < 0.05).

Significant differences in the hydrolysis of WGPs by different proteases can be observed from the above results, which can be attributed to variations in enzymatic properties among these proteases. Protamex, Flavourzyme, and the neutral protease exhibit both endoprotease and peptidase activity, resulting in a higher DH for their respective hydrolysates. ProteAXH has significant peptidase activity and thus achieves the highest DH value. In contrast, the acidic protease and neutral protease only possess endoprotease activity, leading to a lower DH for their corresponding hydrolysates. However, it should be noted that the performance of different proteases in hydrolysis cannot represent their actual ability to degrade WGP due to differences in the initial enzyme activity. Nevertheless, the impact of pretreatment methods on the hydrolysis of specific proteases can be effectively evaluated by comparing their performance in hydrolyzing differently pretreated WGPs.

Furthermore, all three pretreatments induced alterations in the protein structure and an increase in solubility and *H*_0_. Such changes seem to suggest the greater exposure of enzyme cleavage sites in proteins, rendering them more susceptible to binding with proteases and thereby enhancing the enzymatic efficiency. However, our research indicates that the same treatment conditions can yield varying and even contrasting effects on enzymatic hydrolysis depending on the type of protease used. Due to the inherent diversity of the amino acid sequences in proteins [31], there may be variations in sensitivity to the same treatment conditions. This variability can result in differences in flexibility and compactness among different segments. While the secondary structure, *H*_0_, and solubility can provide overall indications of protein structural changes, they cannot capture specific alterations in individual peptide segments. Protein hydrolysis cleavage sites exhibit high sequence specificity; therefore, the modifications occurring within the protease cleavage site exert a significant impact on protease cleavage, rather than inducing any global conformational changes in the protein. Consequently, when applied to different proteases, identical pretreatment conditions may yield diverse outcomes. For instance, AHT inhibits the hydrolysis of ProteAXH and the acidic protease but enhances that of the three other proteases. Thus, no single pretreatment method can effectively promote hydrolysis by all proteases.

#### 3.2.2. TCA-Soluble Peptide Content

**Figure 4:**
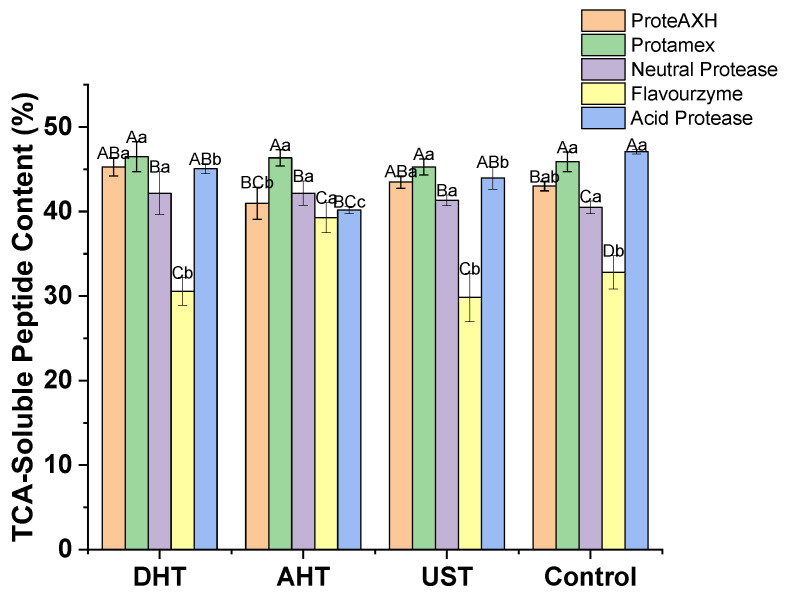
Content of TCA-soluble peptides in the hydrolysates. DHT: dry heating treatment; AHT: aqueous heating treatment; UST: ultrasound treatment. Uppercase letters indicate significant differences within different pretreatments, while lowercase letters indicate significant differences within varied proteases (*p* < 0.05).

The TCA-soluble peptide content in the hydrolysates of WGPs is illustrated in Figure 4. The pretreatment method did not significantly affect the TCA-soluble peptide content of the hydrolysate (*p* > 0.05). However, the protease had a highly significant impact on it (*p* < 0.01). Specifically, the Protamex hydrolysate exhibited the highest TCA-soluble peptide content, while the Flavorzyme hydrolysate showed the lowest. Although there were no significant differences between ProteAXH and the neutral protease as well as the acid protease, there were significant variations in TCA-soluble peptide content among other protease hydrolysates (*p* < 0.05). There was a significant interaction between the pretreatment and protease (*p* < 0.01). For ProteAXH and the acid protease, AHT resulted in the lowest TCA-soluble peptide content; however, for Flavourzyme, the opposite was true, with the highest content obtained from AHT. Protamex consistently demonstrated similar results under all conditions; furthermore, all three pretreatment methods increased the TCA-soluble peptide content in neutral protease hydrolysates.

It is worth noting that while there were substantial variations in DH among the different groups, their content of TCA-soluble peptides remained similar (Figure 3 and Figure 4). The solubility of peptides in TCA solutions cannot accurately indicate their size due to the combined influence of hydrophobicity and the chain length, and the relationship between the solubility and chain length does not strictly follow a negative trend [32]. The single TCA-soluble peptide content and DH alone do not provide an indication of the peptide size. However, the size of peptides can be inferred by combining the results of the TCA-soluble peptide content and DH. This can be attributed to the fact that the DH, determined using the OPA method, quantifies the increase in α-amino groups resulting from enzymatic hydrolysis [23], while the content of TCA-soluble peptides, measured by employing the BCA method, assesses the peptide bonds present in the hydrolysate [33]. Although it cannot completely eliminate potential interference from specific amino acid residues reacting with the BCA reagent on the results, conducting measurements at a higher temperature can enhance the sensitivity towards reactions between reagents and peptide bonds while minimizing the influence of the protein’s amino acid composition on the results [33]. Therefore, under the condition of equal total amino acid content in the hydrolysates, a decrease in the average peptide chain length will result in a reduction in TCA-soluble peptide content. For instance, hydrolyzing a peptide composed of 100 amino acids can result in the formation of either 10 decapeptides or 50 dipeptides. This leads to an increase in the number of α-amino groups from 1 to 10 or 50, while decreasing the number of peptide bonds from 99 to either 90 or 50. Consequently, it changes the ratio of peptide bonds to α-amino groups from 99:1 to either 9:1 or 1:1. Such inferences cannot determine the exact sizes of peptides; however, comparing changes in these two indicators among different groups can help to determine the relative lengths of the peptides in the hydrolysates. The hydrolysates prepared by ProteAXH exhibited high DH but low levels of TCA-soluble peptides, indicating the production of numerous short peptides and/or free amino acids during ProteAXH hydrolysis due to its strong endo- and exo-protease activity. Similar results were found with Flavourzyme hydrolysis. On the other hand, acid protease hydrolysates had low DH values, yet their TCA-soluble peptide content was high, indicating that the acid protease had a limited hydrolysis effect on WGPs and produced larger peptides.

The impact of pretreatment on the length and quantity of generated peptides can also be determined by comparing the changes in TCA-soluble peptides and DH. The hydrolysates prepared using ProteAXH, Flavourzyme, Protamex, and the acid protease showed consistent trends in these two indicators, indicating that the pretreatments did not significantly affect the peptide chain length generated by enzymatic hydrolysis using these proteases. However, the neutral protease demonstrated a significantly higher DH after AHT compared to the other treatment groups; nevertheless, the TCA-soluble peptide content remained similar among all groups. This indicates that AHT can facilitate the production of smaller peptides by a neutral protease.

#### 3.2.3. DPP-IV Inhibitory Activity

**Figure 5:**
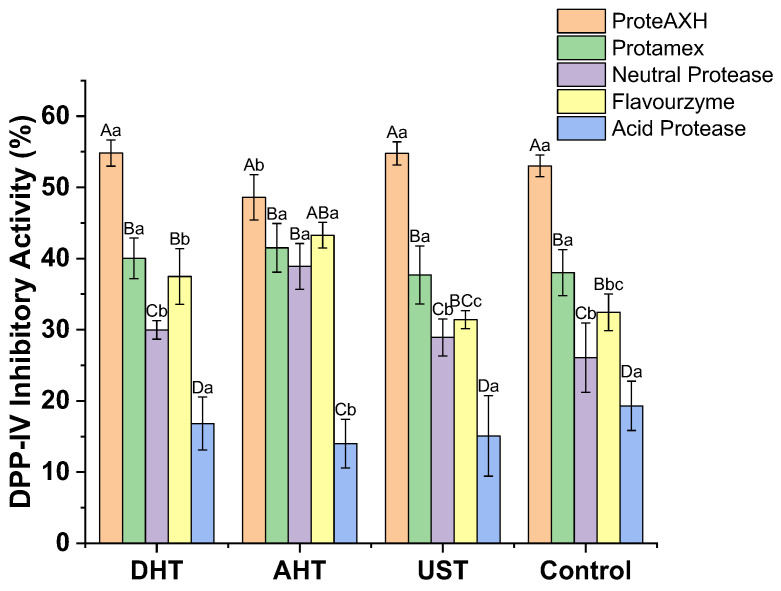
The DPP-IV inhibitory activity of the hydrolysates derived from wheat gluten proteins subjected to different pretreatments. DHT: dry heating treatment; AHT: aqueous heating treatment; UST: ultrasound treatment. Uppercase letters indicate significant differences within different pretreatments, while lowercase letters indicate significant differences within varied proteases (*p* < 0.05).

The results of DPP-IV inhibitory activity for the hydrolysates are presented in Figure 5. Overall, the changes in DPP-IV inhibitory activity exhibit a similar trend to that observed for DH.

The results of the two-way ANOVA analysis revealed a significant effect of the pretreatment on DPP-IV inhibitory activity (*p* < 0.01). AHT exhibited a significantly different level of DPP-IV inhibition compared to UST and the control group (*p* < 0.05). The type of protease had a significant influence on the hydrolysates’ activity (*p* < 0.01). Among the different types of proteases, the ProteAXH hydrolysate showed the highest activity, while the acid protease hydrolysate exhibited the lowest activity. Except for Flavourzyme and Promatex, which did not have a significant impact on activity, there were notable differences in DPP-IV inhibition among the various types of proteases (*p* < 0.05). There was also a significant interaction between the pretreatment and protease regarding their effects on DPP-IV inhibitory activity in the hydrolysates (*p* < 0.01), indicating that their combination had an observable effect.

There was no significant difference observed between AHT and DHT in terms of their effects on activity; however, for specific proteases, these two pretreatment methods may have opposite effects. For instance, AHT demonstrated evident inhibitory effects on ProteAXH and the acid protease hydrolysates’ DPP-IV inhibitory activity but exhibited promoting effects in the neutral protease and Flavourzyme cases (*p* < 0.05). The Protamex hydrolysate’s activity did not show any noticeable differences under the four treatment conditions.

As previously mentioned, the variation in the initial activity of proteases is one of the significant reasons for differences in the DH of hydrolysates, which also applies to DPP-IV inhibitory activity. Additionally, the amino acid composition is not the sole determinant of DPP-IV inhibitory activity, as the size of the peptides also exerts a substantial influence. Generally, smaller peptide fractions display higher DPP-IV inhibitory activity [34]. The findings of this study support this inference, as ProteAXH hydrolysates, which contained a higher proportion of short peptides, exhibited the most potent DPP-IV inhibitory activity. In contrast, peptides generated by the acid protease with longer chain lengths exhibited lower DPP-IV inhibitory activity.

The above results demonstrate that the pretreatment method significantly influences the generation of DPP-IV inhibitory peptides, with its effects strongly depending on the type of protease used. Among the various pretreatments in WGP hydrolysis, AHT was found to have a significant inhibitory effect on DPP-IV inhibitory peptide production by ProteAXH and the acid protease, while greatly improving the preparation when using Flavourzyme, Protamex, and the neutral protease. To gain a better understanding of the influence of the pretreatment and protease on the processing properties of DPP-IV inhibitory peptides, hydrolysates derived from three proteases (ProteAXH, Flavourzyme, and Protamex) were selected for further study.

### 3.3. Processing Properties of the Hydrolysates

#### 3.3.1. Foaming Properties

The foaming properties play a crucial role in the field of food processing as they contribute to the formation of a soft texture and enhanced mouthfeel [24,35]. Foams consist of air dispersed in a continuous liquid, which makes them thermodynamically unstable [36]. The foaming capacity (FC) and foam stability (FS) are usually used to assess the foaming performance. Table 2 presents the foaming properties of the hydrolysates from WGPs.

The effects of both pretreatment methods and different types of proteases on the foaming properties were investigated, revealing their significant impacts on the foaming capacity (FC) of hydrolysates (*p* < 0.01), along with a notable interaction between these two factors. Among the three tested proteases, hydrolysates prepared using Flavorzyme demonstrated remarkably high FC, ranging from 238.67% to 252.00%, whereas those produced by ProteAXH exhibited the lowest FC (106.67~139.00%). There was also a significant interaction between the protease type and pretreatment method on the FC (*p* < 0.01). Although UST was found to promote an increase in FC according to the analysis results (*p* < 0.01), AHT and DHT were observed to decrease it significantly (*p* < 0.01). However, it should be noted that the specific protease used has a significant impact on the FC of the hydrolysate.

The FC of hydrolysates was influenced by the pretreatment method in a protease-dependent manner. In ProteAXH hydrolysis, DHT and UST increased the FC by 11.18% and 22.65%, respectively, while AHT slightly decreased it by 5.88%. All three treatments resulted in a decrease in FC during Protamex hydrolysis (ranging from 1.68% to 16.71% compared to control), while they slightly improved the FC during Flavourzyme hydrolysis (ranging from 2.79% to 5.59%).

Similarly, the FS of hydrolysates is significantly influenced by the pretreatment method and type of protease (*p* < 0.01). Among the different proteases, Protamex yielded hydrolysates with the highest FS, while Flavourzyme resulted in the lowest FS (*p* < 0.01). The impact of the pretreatment methods varied depending on the specific protease used. When ProteAXH was employed for hydrolysis, any pretreatment led to a decrease in the FS of the hydrolysate. DHT enhanced the FS in Promatex hydrolysates, whereas AHT and UST had inhibitory effects. Hydrolysates produced by Flavourzyme achieved the highest FS after undergoing UST.

The hydrolysates produced from Protamex or Flavourzyme showed outstanding FC in line with previous findings that proteolysis could significantly enhance the FC of the protein [37,38]. This can be attributed to their remarkable ability for rapid transportation, absorption, and rearrangement at the air–water interface [39,40]. However, hydrolysates prepared by ProteAXH showed poor FC due to the extensive hydrolysis of WGPs into short peptides with low molecular weights. This is because a protein hydrolysate’s FC depends on its molecular weight or peptide length [41]. It has been proven that low-molecular-weight peptides are insufficient to maintain a well-ordered molecular orientation at the interface [39,40].

#### 3.3.2. Emulsion Activity

Emulsion activity (EA) is a property exhibited by amphiphilic substances, similar to the foaming properties. When subjected to vigorous stirring, an oil can disperse in an aqueous solution and create substantial interfacial tension at the oil–water interface. As shown in Table 2, both the pretreatment and proteases had significant effects on EA (*p* < 0.01). The EA of ProteAXH hydrolysates was the lowest (<10%), whereas that of Protamex hydrolysates exhibited a significantly higher level compared to the other two proteases (*p* < 0.01). This disparity can be attributed to the inhibitory effect caused by smaller-sized peptides, which hinder the proper maintenance of a well-ordered interface orientation for the molecule [41]. DHT resulted in significantly higher EA for both Protamex and Flavourzyme compared to other pretreatment methods (*p* < 0.01). There was a notable interaction between the pretreatment methods and types of proteases. Pretreatment substantially improved the EA for Flavourzyme, increasing it from 1.98% to 52.96% (*p* < 0.01), whereas, for Protamex, AHT lead to nearly a 50% decrease (*p* < 0.01), while there were no statistically significant differences between DHT, UST, and the control group.

In summary, the WGP hydrolysates were a mixture of various peptide segments. The size, *H*_0_, flexibility, and surface charges of the peptides differed depending on the pretreatment and protease used. The variations in these properties led to differences in the foaming and emulsifying properties of the hydrolysates [42,43,44].

## 4. Conclusions

In general, the impact of different pretreatment methods on the structure and physicochemical properties of WGP varies. The type of protease exerts a more significant influence on WGP hydrolysis and the resulting hydrolysates’ processing properties compared to the pretreatment method used. Depending on the specific protease employed, certain pretreatment methods can either enhance or inhibit protein hydrolysis. Furthermore, both the pretreatment methods and types of protease interactively influence both the hydrolysis of WGP and the processing properties of the resulting hydrolysates.

## Figures and Tables

**Table 1:** Secondary structure content of natural and pretreated wheat gluten proteins (mean ± standard deviation).

Pretreatment	β-Sheet (%)	α-Helix (%)	β-Turn (%)	α-Helix/β-Sheet
Control	50.89 ± 0.02 ^c^	26.33 ± 0.14 ^b^	22.78 ± 0.14 ^b^	0.52 ± 0.00 ^b^
DHT	49.82 ± 0.29 ^d^	26.88 ± 0.11 ^a^	23.30 ± 0.18 ^a^	0.54 ± 0.01 ^a^
AHT	52.09 ± 0.31 ^a^	25.33 ± 0.08 ^d^	22.58 ± 0.23 ^b^	0.49 ± 0.00 ^d^
UST	51.36 ± 0.22 ^b^	26.00 ± 0.18 ^c^	22.64 ± 0.04 ^b^	0.51 ± 0.01 ^c^

DHT: dry heating treatment; AHT: aqueous heating treatment; UST: ultrasound treatment. The letters represent significant differences (*p* < 0.05) in the content of secondary structures of proteins.

**Table 2:** Processing properties of the hydrolysates (mean ± standard deviation).

Protease	Pretreatment Method	Foaming Capacity (%)	Foam Stability (%)	Emulsion Activity (%)
ProteAXH	DHT	126.00 ± 1.73 ^Fb^	10.07 ± 1.96 ^Ec^	<10
	AHT	106.67 ± 2.89 ^Gc^	10.89 ± 2.37 ^Ec^	<10
	UST	139.00 ± 3.61 ^Ea^	16.52 ± 1.50 ^Db^	<10
	Control	113.33 ± 7.64 ^Gc^	23.43 ± 2.17 ^Ca^	<10
Protamex	DHT	214.33 ± 4.04 ^Db^	35.15 ± 2.78 ^Aa^	49.82 ± 0.30 ^Ba^
	AHT	216.00 ± 5.29 ^Db^	10.51 ± 1.31 ^Ed^	26.67 ± 1.61 ^Eb^
	UST	253.00 ± 5.57 ^ABa^	20.95 ± 0.31 ^Cc^	51.40 ± 0.80 ^Ba^
	Control	257.33 ± 0.58 ^Aa^	31.09 ± 0.83 ^Bb^	51.05 ± 0.53 ^Ba^
Flavourzyme	DHT	245.67 ± 4.04 ^BCb^	17.23 ± 0.34 ^Da^	54.21 ± 1.39 ^Aa^
	AHT	252.00 ± 3.46 ^ABa^	9.13 ± 0.76 ^Eb^	38.60 ± 1.22 ^Cb^
	UST	245.33 ± 2.52 ^BCb^	10.20 ± 2.09 ^Eb^	36.14 ± 0.61 ^Dc^
	Control	238.67 ± 1.15 ^Cc^	9.92 ± 1.39 ^Eb^	35.44 ± 1.22 ^Dc^

DHT: dry heating treatment; AHT: aqueous heating treatment; UST: ultrasound treatment. The uppercase letters indicate significant differences among all test samples, while lowercase letters indicate significant differences among the hydrolysates prepared using the same protease (*p* < 0.05).

## Data Availability

Data are contained within the article.
